# Plant Extracts and SARS-CoV-2: Research and Applications

**DOI:** 10.3390/life13020386

**Published:** 2023-01-31

**Authors:** Sandrina A. Heleno, Marcio Carocho, Filipa S. Reis, Tânia C. S. P. Pires, Manuela Pintado, Isabel C. F. R. Ferreira, Lillian Barros

**Affiliations:** 1 Centro de Investigação de Montanha (CIMO), Instituto Politécnico de Bragança, Campus de Santa Apolónia, 5300-253 Bragança, Portugal; 2 CBQF—Centro de Biotecnologia e Química Fina—Laboratório Associado, Escola Superior de Biotecnologia, Universidade Católica Portuguesa, Rua de Diogo Botelho 1327, 4169-005 Porto, Portugal

**Keywords:** SARS-CoV-2, COVID-19, pandemic, secondary metabolism, anti-viral agents

## Abstract

The recent pandemic of COVID-19 caused by the SARS-CoV-2 virus has brought upon the world an unprecedented challenge. During its acute dissemination, a rush for vaccines started, making the scientific community come together and contribute to the development of efficient therapeutic agents and vaccines. Natural products have been used as sources of individual molecules and extracts capable of inhibiting/neutralizing several microorganisms, including viruses. Natural extracts have shown effective results against the coronavirus family, when first tested in the outbreak of SARS-CoV-1, back in 2002. In this review, the relationship between natural extracts and SARS-CoV is discussed, while also providing insight into misinformation regarding the use of plants as possible therapeutic agents. Studies with plant extracts on coronaviruses are presented, as well as the main inhibition assays and trends for the future regarding the yet unknown long-lasting effects post-infection with SARS-CoV-2.

## 1. Origin and Evolution of Pathogenic Coronaviruses

Until 2003, when the severe acute respiratory syndrome (SARS-CoV-1) appeared in China, coronaviruses were not considered dangerous to humans; they mostly caused infections in people with compromised immune systems. The Middle East respiratory syndrome coronavirus (MERS-CoV) appears ten years later in Middle Eastern countries [1]. In 2019, SARS-CoV-2, previously known as 2019-nCoV, emerged in Wuhan, China, and then spread worldwide, creating a global pandemic, named COVID-19, the likes of which had not been felt around the globe since the Influenza Pandemic (Spanish flu) of 1918.

SARS-CoV-1, which first emerged in Foshan, Guangdong, China, was responsible for a viral pneumonia from 2002 to 2004 and killed at least 770 individuals of the approximately 8000 it infected in 30 countries, showing a fatality rate of approximately 10%. The outbreak is believed to have started somewhere in the food chain, namely among workers in this industry. The symptoms were characterized by a fever followed by respiratory problems, which would progress to respiratory failure. The main difference, perceived at the time, between SARS-CoV and other infectious outbreaks was the hospital rupture it could cause.

In contrast, other people remained relatively healthy and could spread the disease across borders [2,3]. While China was quite restrictive in allowing news about the causative agent, its reports attributed the outbreak to *Chlamydia*. Still, by March 2002, laboratories throughout the world had found the culprit through polymerase chain reaction (PCR), detecting the first pathogenic strain of coronaviruses in humans.

The most accepted hypothesis for the spillover to humans claims that SARS-CoV spread from bats of the Hipposideridae family (natural reservoir) to the bats from the Rhinolophidae family, then to masked palm civets, and finally to humans [2,3,4].

MERS-CoV was incidentally discovered in Saudi Arabia in 2012, in a fatal human case of viral pneumonia, which affected over 1300 human cases, with a fatality rate of around 35%. MERS is also known as the camel flu because it also infects these animals, although bats seemed to be the original reservoir of this strain. This outbreak was characterized by a higher mortality rate, with the symptoms being quite similar to SARS-CoV-1. However, a progression to acute kidney failure was quite common, as well as a specific lower respiratory infection [5]. MERS was the second pathogenic strain of coronavirus that could infect humans.

The year 2019 marked the emergence of a third coronavirus and a consequent pandemic that had not occurred in the previous two outbreaks. In a wet seafood market in Wuhan, Hubei, China, a cluster of pneumonia cases seems to be the outbreak’s origin, following a spillover from bats to humans, highly related to SARS-CoV, officially named SARS-CoV-2. This new coronavirus showed a higher transmission rate and a more extended asymptomatic incubation period, which led to higher infectiousness [6]. In December 2021, SARS-CoV-2 had reached over 290 million confirmed cases and killed over 5 million people, while at the end of 2022, the cumulative number of confirmed cases had risen to 645 million cases and been fatal for over 6.5 million [7].

## 2. Morphology and Infection Route of Coronavirus

Coronaviruses belong to the Coronaviridae family, consisting of four genera, namely, *alpha*, *beta*, *gama* and *delta* coronavirus; *alpha* and *beta* genera being the ones that infect. Until 2019, SARS-CoV and MERS-CoV were the most highly contagious coronaviruses, while the other four were responsible for the common colds (HCoV-OC43, HCoV-229E, HCoV-NL63 and HCoV-HKU1) [1,8].

**Figure 1:**
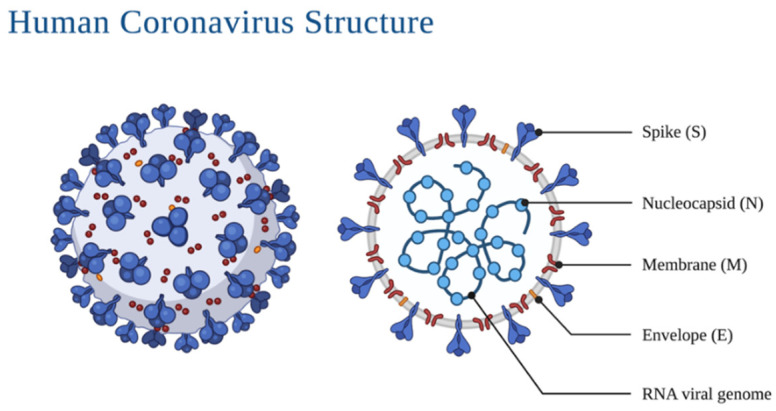
Detail of the SARS-CoV-2 virus and different aspects of its morphology.

**Figure 2:**
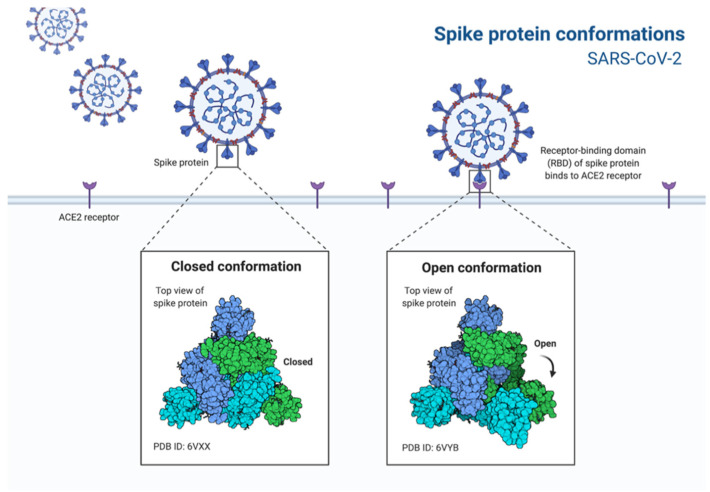
Depiction of the conformation change in the spike protein of SARS-CoV-2 to bind to the cell ACE2 receptors.

Morphologically, coronaviruses display three protein structures on their surface: the spike glycoprotein, the viral membrane glycoprotein, and the envelope protein (Figure 1). The spike protein mediates the entrance into the cell through the host’s angiotensin-converting enzyme 2 (ACE2) (Figure 2) surface receptor; the membrane glycoprotein is unique compared to other glycoproteins in its features making it responsible for its intracellular budding. The envelope protein is involved in the virus assembly, budding and pathogenesis [9,10]. The spikes present on the virus’ surface make it resemble a crown, ergo, the name corona (crown in Spanish). Its genome ranges between 26 and 32 kb, with 6–11 open reading frames that encode 9680 amino acid polyproteins.

Specific genes in the open reading frame (ORF1) downstream regions, which encode proteins for viral replication, nucleocapsid and spikes formation, are present in all coronaviruses. The RNA is uncoated to allow translation of the two polyproteins, transcription of the subgenomic RNAs and replication of the viral genome. Formed envelope glycoproteins are inserted in the Golgi membranes; genomic RNA and nucleocapsid proteins combine to form nucleocapsids, and the viral particles bud into the ER–Golgi intermediate compartment. Virion-containing vesicles subsequently fuse with the plasma membrane to release the virus [11].

Downregulation of ACE2 and the binding of the spike protein of this receptor contribute to lung injury during SARS, resulting in the excessive production of angiotensin II, stimulating the type 1a angiotensin II receptor (AGRT1A), increasing the pulmonary vascular permeability and explaining the increased lung pathology, with decreased expression of ACE2 [11].

Coronavirus entry into host cells is dependent on the binding of the spike glycoprotein to the cellular receptor. SARS-CoV and SARS-CoV2 use the host’s ACE2 protein, which usually breaks down angiotensinogen and regulates blood pressure and inflammation, as a doorway to enter the cell by binding to it and sneaking into the host cell, many by fusion and endosomal pathway entry. Due to a high number of ACE2 receptors in the lungs, the coronavirus mainly and initially infects the lung tissue, but can spread to several organs due to most tissues being lined with ACE2. After the entrance, viral RNA is released into the host’s cytoplasm and undergoes translation, generating both genomic and subgenomic RNA. The virion assembly occurs in the endoplasmic reticulum and the Golgi complex and is then released through vesicles [9,12].

## 3. Antiviral Therapies and Vaccine Strategies

When the COVID-19 pandemic reached global proportions, considerable private and public funds were poured into the development of vaccines and antiviral drugs. While vaccines were still at least a year away from being produced on a large scale, which only occurred at the end of 2020 many other drugs were proposed (Table 1). As the pandemic rose in severity during the year of 2021 with new variants, so did the quest to find suitable existing drugs and new ones to fight back in severe disease cases.

In September 2021, the European Medicines Agency (EMA) has only one authorized drug against COVID-19, remdesivir, sold under the brand name of Veklury^®^, while three marketing authorization applications had been submitted, namely, Olumiant^®^ (baricitinib), Kineret^®^ (anakinra) and RoActemra^®^ (tocilizumab), all of which are already authorized for other illnesses within the EU. The EMA also stated that four medicines are under rolling review, namely, Bamlanivimab and Etesevimab, Regdanvimab, REGN-COV2^®^ (casirivimab/imdevimab) and Sotrovimab (December 2021). The Food and Drug Administration of the United States of America (FDA) states on their webpage the following “Drug and Biological Therapeutic Products” with emergency approval: Actemra^®^ (tocilizumab), Sotrovimab^®^, Bamlanivimab and Etesevimab^®^, REGEN-COV^®^ (Casirivimab and Imdevimab), Olumiant^®^ (barcitinib), COVID-19 convalescent plasma and remdesivir. Other drugs have been proposed to treat COVID-19 but have not been effective in showing any improvements in patients, namely, hydroxychloroquine, lopanivir/ritonavir and convalescent plasma [13,14,15]. 

**Table 1:** Approved, in analysis and permission-sought drugs in the EU and US against COVID-19 (September 2021).

Rug Name	Main Function	Permission Sought	Reference
Veklury^®^ (remdesivir)	After conversion to remdesivir monophosphate, this compound stalls viral RNA-polymerase	EU and US	[16]
Olumiant^®^ (baricitinib)	This tyrosine kinase regulates the immune response of the body, avoiding a cytokine storm	EU and US	[17]
Kineret^®^ (anakinra)	This recombinant interleukin (IL)-1 receptor antagonist helps control the hyperinflammatory syndrome of COVID-19	EU	[18]
RoActemra^®^ Actemra^®^ (tocilizumab)	Recombinant humanized monoclonal antibody that prevents the binding of IL-6 to its receptor, inhibiting the inflammatory cascade	EU and FDA	[19]
Bamlanivimab and etesevimab	Neutralizing monoclonal antibodies that target the spike glycoprotein of the virus rendering it impossible to enter the host cell	EU and US	[20]
Regdanvimab	Monoclonal antibodies which also reduce viral entry in host cells	EU	[21]
REGN-COV-2^®^ Casirivimab/imdevimab	Monoclonal antibodies that also target the spike glycoprotein	US	[22]
Sotrovimab^®^ (VIR-7831)	Monoclonal antibodies that reduce viral entry into host cells and help clear infected cells	EU and US	[23]

The main therapies against COVID-19 are antiviral drugs and monoclonal antibodies, which have shown some improvements in moderate and severe diseases. Still, some resistance has been found to some drugs, namely, the monoclonal antibodies Bamlanivimab and etesevimab, derived from mutations in the spike glycoprotein that changes its conformation, rendering the antibodies less effective [24].

However, the most effective method of controlling the pandemic has been proven to be through vaccines (Table 2).

Among the existing vaccines, there are four different technologies. One of the most effective is the mRNA vaccines, which use a strain of RNA from the coronavirus, which instructs the human cells how to build the spike protein that is then recognized by the immune system, creating antibodies to fight it [25]. This technology is used by the approved Comirnaty and Spikevax vaccines, and the CureVac, which is still under rolling review. Another technology is the vector vaccine, used by the approved Vaxzevria and Janssen, in which a portion of the genetic material of the coronavirus is placed in a different virus (viral vector), usually an adenovirus, which when inside human cells releases the viral load and instructs the cells to make copies of the spike glycoprotein. This technology is also used by the Sputnik V vaccine [26]. The third technology used in the approved and rolling review vaccines is the protein subunit technology which includes only parts of the coronavirus’ RNA, namely, the spike glycoprotein, which when inside the cells makes the host’s immune system recognize these proteins and create antibodies. Novavax and Sanofi use this technology in their vaccines. Finally, Sinopharm uses the classical approach in terms of vaccines, opting to inject the whole SARS-CoV-2 virus in an inactivated form, allowing the immune system to build up defenses against this virus [26].

## 4. Plant Metabolites: Debunking Misconceptions

The COVID-19 pandemic showed that even in the face of worldwide unrest, millions of infections and deaths, certain denier movements and organizations were still questioning the pandemic, its origins, and, in some cases, proposing homemade remedies and questionable medications to fight the virus or infection symptoms. These groups were fueled by misinformation mainly spread through the Internet, specifically social media [27]. Among the proposed remedies, many of them based on empirical knowledge, using simple logic, it was assumed that if the symptoms of COVID-19 were those of the common flu then the same homemade remedies could help and cure the infection. These remedies and Chinese medicine-based papers have been published [28,29,30,31,32,33,34] alongside others that claim that specific vitamins and other remedies may improve the recovery or boost the immune system against SARS-CoV-2, but not without controversy [35,36,37,38]. One example of these misconceptions was the use of chloroquine and hydroxychloroquine (antimalarial drugs) against covid, which after much research was deemed to not have any positive effect on overall mortality, ventilation needs and hospitalization, despite promising results in vitro. Thus, it remains to be confirmed if it has any effect on the early stages of the disease [39,40], and one report event accounts for a worsening of the clinical status [41]. Another proposed drug that was proposed as a treatment for COVID-19 was ivermectin, an antiparasitic treatment. Several publications have also debunked its use, claiming that the drug did not result in a lower incidence of admission to hospitals and prolonged emergency observation [42]. Furthermore, in another clinical trial, neither ivermectin or metformin and fluvoxamine showed effects on hypoxemia, hospitalization or death of patients with COVID-19 [43].

While there is generalized scientific support that plants and their metabolites may play a role in drugs against COVID, its prophylaxis, or be used as active agents in fomites or textiles, the approach to using natural molecules should be the same as any other molecule. A big misconception present in most societies is that natural molecules are always safer, more effective, and sustainable, although this is not always the case [44].

**Figure 3:**
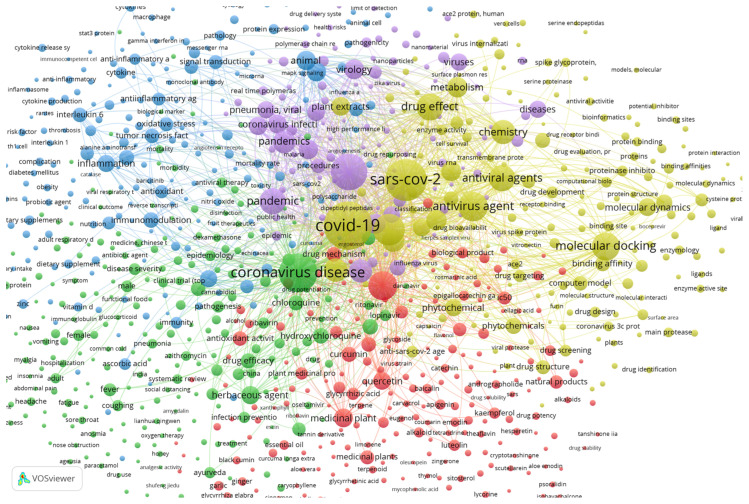
Plot from VOSviewer showing the bibliographic data from specific keywords between 2020 and 2022, totaling 1504 documents grouped by co-occurrence.

Using the SCOPUS database and VOSviewer software, a brief relation between plants and SARS-CoV-2 can be established. About 1504 documents show up using the keywords “coronavirus”, “covid”, “sars”, “plant” and “extract” from 2020 to 2022 within the SCOPUS search engine. Documents that contain at least one of the searched keywords were clustered by VOSviewer according to their relevance and relationship. The plot of the relationships between the documents with these keywords is shown in Figure 3.

Figure 3 was obtained by analyzing the co-occurrence of bibliographic data obtained from the previously mentioned keywords. The relatedness of items is determined based on the number of documents in which they occur together, with a minimum of 5 occurrences of a keyword to be considered. The visualization weight was set at the total link strength and showed a maximum of 1000. Regarding the relationship between the keywords and plants and metabolites, some specific compounds have connections with other keywords, showing that considerable amounts of studies used those compounds as potential antiviral candidates.

**Figure 4:**
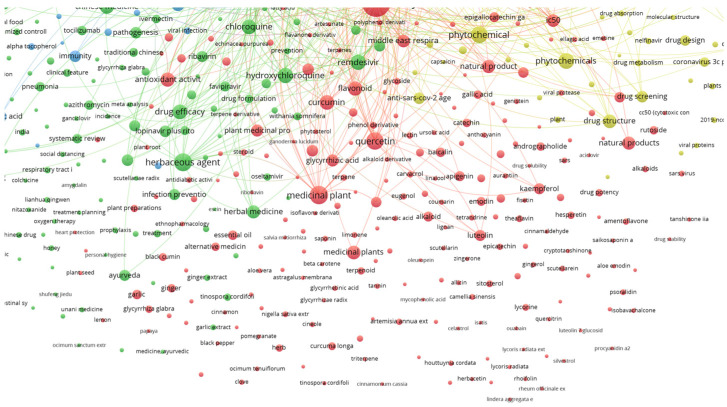
Detail of relationships between plant secondary metabolites and the searched keywords.

The lines connecting the keywords show a strong connection between them, while isolated circles mean that although the keywords occur together, there are fewer documents with both, implying a lower correlation between them, and thus these compounds have less importance. This can also be observed by the physical distance between the circles. Examples of compounds highly related to the defined keywords and with connecting lines are quercetin, luteolin, kaempferol, carvacrol and gallic acid, shown in Figure 4.

While specific metabolites do occur in Figure 4, there could be many others that are still under research and have not been published yet. The time frame set to build Figure 3 and Figure 4 was set between 2020 and 2022, which is a very short period considering the time the research takes to achieve the results, followed by the writing and publication of the study. Still, the fact that some individual compounds already show bibliographic correlation is impressive and reveals that there is potential. Furthermore, keywords such as medicinal plant, flavonoid, phytochemicals, natural product, flavanone derivative and phytotherapy are all correlated with the keywords, but do not reveal the specific used metabolite or extract, thus demanding further rummage and increasing the potential of natural products in fighting SARS-CoV-2.

## 5. Antiviral Activity of Natural Sources

Nature has proven to be an inexhaustible source of molecules and compounds for several health products, namely, drugs, remedies and other related formulations [45]. Among the most well-known and studied compounds with biological activity obtained from natural products, the phenolic compounds stand out as one of the classes with higher potential, including as natural antivirals. Table 3 summarizes some of the available information regarding the antiviral activity attributed to them observed in vitro. There are no apparent specific subgroups with higher activity than others; thus, the chemical structure may influence their bioactivity. For example, some extracts rich in phenolic glucosides, obtained from the poplar tree cultivar Beaupré (*Populus trichocarpa* Torr. & A. Gray ex. Hook.), were effective against the Poliomyelitis virus or the Semliki Forest virus [46], and the presence of free hydroxyl and ether groups seems to influence the anti-rabies activity of phenolic compounds. Natural extracts obtained from rose root (*Rhodiola rosea* L.) aerial parts revealed the presence of gallic and ellagic acids, two structurally related compounds, being identified as Ebola virus entry inhibitors [1]. In turn, the aqueous extracts from Indian madder (*Rubia cordifolia* L.) aerial parts showed anti-viral effects against rotavirus. Sun et al. [47] identified xanthopurpurin and vanillic acid as the main ones responsible for the anti-viral effect against the human immunodeficiency virus. Some HIV-related studies have also shown that kaempferol, and mainly kaempferol-7-*O*-glucoside, extracted from *Securigera securidaca* (L.) Degen & Dörfl., could be considered as a new potential drug candidate for the treatment of HIV infection [48].

There are also several studies describing the bioactivity of phenolic extracts against the herpes virus (Table 3). Extracts rich in gallic acid (phenolic acid), apigenin (flavone), and naringin (flavanone-7-*O*-glycoside) appear to be effective against the herpes simplex virus type 1 (HSV-1) [49,50]. Other phenol derivatives such as *p*-quinone monooximes (obtained from the basic nitrosation of 3-methoxyphenol), and 8-hydroxyquinoline also showed effects in vitro against HSV-1 [51].

Phenolic compounds have also been shown to have antiviral potential against viruses that affect the respiratory system (Table 3). Özçelik et al. [49], verified that an alkaloid called atropine (commercially available) has a remarkable inhibitory effect in vitro against parainfluenza virus type 3 (PI-3), while also being effective against HSV-1. The authors confirmed that genistein (isoflavone), and the phenolic acids gallic, chlorogenic, and quinic, exerted varying degrees of anti-PI-3 effects. Some fractions isolated from the ethanol extract from *Origanum vulgare* L. also revealed some activity against the respiratory syncytial virus (RSV). However, this activity was weak to moderate when compared with positive controls (commercial drugs).

The compounds identified in these fractions were apigenin and a flavone glycoside identified as acacetin 7-*O*-[4‴-*O*-acetyl-β-d-apiofuransyl-(1→3)]-β-d-xylopyranoside [50]. Extracts obtained from the flowers of *Bombax ceiba* L., particularly rich in the flavonoids quercetin and kaempferol-3-*O*-(6″-*O*-E-*p*-coumaroyl)-β-d-glucopyranoside, and the xanthonoid mangiferin, also revealed anti-RSV activity in vitro [52].

Still within the topic of respiratory viruses, several studies have been carried out to verify the effect of natural extracts rich in phenolic compounds in influenza viruses. Nile et al. [53] isolated quercetin 3-glucoside from fringed pink (*Dianthus superbus* L.) leaves, performing tests in vitro and in silico that revealed this flavonoid might act as a natural anti-influenza drug. Takeda et al. [54] analyzed the antiviral activity of hibiscus (*Hibiscus sabdariffa* L.) tea extract against the human Influenza A virus (IAV). The authors evaluated its potential as a novel anti-IAV drug and a safe inactivating agent for a whole inactivated vaccine. The in vitro study revealed that the pH of the hibiscus tea extract is acidic, and its rapid and potent antiviral activity depends mainly on the acidic pH. Given this characteristic, the extract is not suitable for therapeutic or vaccination purposes. However, given the potential of hibiscus tea extract and protocatechuic acid, one of the main components of the extract, with potent acid-dependent antiviral activity, the authors suggest further study of the low-pH-independent antiviral mechanism and attempts to enhance the antiviral activity may establish a novel anti-IAV therapy and vaccination strategy. In another study, You et al. [55] verified that the phenolic compounds present in a fraction of the aqueous extract from the tender leaf of *Toona sinensis* (A. Juss.) M.Roem., TSL-1, might have antiviral activities against pandemic influenza A (H1N1) through the downregulation of adhesion molecules and chemokine to prevent viral attachment. The authors demonstrated that catechin might be a safe molecule for long-term use to prevent influenza A (H1N1) virus infection. On the other hand, gallic acid might be a sensitive substance to inhibit influenza virus infection.

**Table 3:** Phenolic compounds antiviral activity; compound or class of compounds, type of compound or extract, extraction methodology and target virus.

Phenolic Compounds/Class	Type of Compound/Extract	Extraction Methods	Virus	Reference
Phenolic glucosides	Ethanol extract from the leaves of the poplar tree cultivar Beaupré (*Populus trichocarpa*)	Ethanol extraction of the hot water-soluble portion followed by polyamide chromatography employing step-gradient elution with water and dilutions of ethanol	Poliomyelitis virusSemliki forest virus	[46]
*p*-Quinone monooximes derived from 3-methoxyphenol - 8-hydroxyquinoline	Synthetic aromatic nitro compounds	Extract washed with brine, dried over magnesium sulfate and filtered. Filtrate evaporated to dryness under reduced pressure. *i*-AmNO_2_ added to a stirred solution of a phenol in DMF in the presence of K_2_CO_3_ at 0 °C under argon. Recrystallization of the crude product from an appropriate solvent gave a *p*-quinone monooxime	Herpes simplex virus type 1 (HSV-1)	[51]
Alkyl-esters of gallic acid 3,4,5-trihydroxy derivatives of gallic acid 3,4,5-trimethoxy derivatives of gallic acid Catechin Epicatechin Quercetin Epigalocatechin	Commercial standard and lab synthesis	-	Rabies virus	[47]
Apigenin Naringin Atropine Genistein Gallic acid Chlorogenic acid Quinic acid	Commercial standard	Compounds dissolved in dimethyl sulfoxide to prepare a final concentration of 256 μg/mL	Herpes simplex virus type 1 (HSV-1) Parainfluenza virus type 3 (PI-3)	[49]
Apigenin Acacetin 7-O-[4′′′-O-acetyl-β-d-apiofuransyl-(1→3)]-β-d-xylopyranoside	*Origanum vulgare* L. plant extracted with 95% (*v*/*v*) ethanol	Air-dried plants percolated with 95% ethanol solution. Ethanol extract concentrated in vacuum to yield a residue, which was suspended in water and partitioned with petroleum ether and ethyl acetate, respectively	Herpes simplex virus type 1 (HSV-1) Respiratory syncytial virus (RSV)	[50]
Kaempferol Kaempferol-7-*O*-glucoside	Hydromethanolic extracts (98%) from *Securigera securidaca* seeds	Dried seeds extracted methanol (98%) at 40 °C. The methanol extract was eluted with *n*-hexane: acetone and then with 100% methanol	HIV-1	[48]
Quercetin Kaempferol-3-*O*-(6″-O-E-p-coumaroyl)-β-d-glucopyranoside Mangiferin	*Bombax ceiba* L. Flowers extracted with 95% (*v*/*v*) ethanol	Extraction by reflux with 95% ethanol	Respiratory syncytial virus (RSV)	[52]
Xanthopurpurin (1,3-dihydroxy-9,10-anthracenedione) Vanillic acid (4-hydroxy-3-methoxybenzoic acid)	Aqueous extract from *Rubia cordifolia* L. aerial parts	Plant aerial parts boiled in distilled water for 1 h, the aqueous solution collected and the residual part re-extracted several times	Rotavirus	[47]
Catechin Gallic acid	Commercial standards	-	H1N1 Influenza virus	[55]
Gallic acid Ellagic acid	Aqueous and organic-solvent extracts of *Rhodiola rosea* L. plant	Extracts dried in vacuum at 50 °C and dissolved in DMSO	Ebola virus	[1]
Quercetin 3-glucoside	Hydromethanolic (70% methanol) and methanol (100%) extracts from *Dianthus superbus* L. leaves	Dried leaves extracted using 70% and 100% methanol	H1N1 Influenza	[53]
Hibiscus acid Protocatechuic acid	Acidic hibiscus tea extract	Hibiscus tea powder soaked in ultrapure water at 24 °C for 24 h, repeating the process several times	H1N1 Influenza virus	[54]

Given the evidence of the antiviral effects of phenolic compounds, several studies have emerged regarding the effects of these compounds against SARS-CoV-2. Table 4 reports some of the tests developed specifically for the SARS coronavirus, showing the particularities mentioned above (compound or class of compounds, type of extraction and extraction methods) and the effective extract/compound concentration. While natural compounds abound, the requisite for finding suitable compounds is quite cumbersome.

These compounds must be widely available, easy to obtain in high quantities, have exceptional efficacy (above-synthesized counterparts) and be easy to isolate and synthesize (due to the pharmaceutical industry preferring synthesized compounds due to higher purity). While these traits are difficult to find in compounds, there are some examples of drugs made from plants that cannot be synthesized, or their synthesis is still too expensive to become commercially viable, even though theoretically, it is possible to synthesize all bioorganic molecules. An example of a molecule initially extracted from nature and then synthesized is Taxol, a potent anticancer drug whose structure was finally elucidated in 1994, allowing its use against cancer [56]. Nevertheless, even after complete compound synthesis is achieved, it may not be commercially viable. The time from its discovery and elucidation of the chemical structure until its commercial success becomes an academic effort with the probability of finding analogs of the original drug with equal or better effects. For example, Halichondrin B, a macrolide with antitumor activity obtained from a marine sponge, was discovered in 1986. However, the molecule’s structure and consequent total synthesis were achieved in 1992. The long period in which these tests took place, and the low synthesis yields, led to the discovery of an analogous compound, Eribulin, with much higher yields [57]. From 1981 to 2010, of all new drugs discovered, 28% were modified natural products, 30% mimicked natural products, and 6% were natural products. Furthermore, 64% of the new pharmaceuticals that entered the market were related to natural products, showing the weight that these molecules can have [58].

The entry steps of the SARS-CoV-2 virus into host cells encompass the attachment of the spike (S) glycoprotein to its receptor, angiotensin-converting enzyme 2 (ACE2), and subsequent membrane fusion. Thus, one of the strategies to inhibit the virus’s entry is finding compounds that can bind the S protein, preventing membrane attachment. Yi et al. [59] identified two small molecules from Chinese herbs with this capacity, tetra-*O*-galloyl-β-D-glucose (TGG) and luteolin. TGG was 50% effective in the inhibition assays with a concentration of 4.5 μM and a selective index of 240.0. On the other hand, luteonin was 50% effective with a concentration of 10.6 μM and a selective index of 14.62. The screening method developed by the authors not only identified these two compounds but also allowed several small molecules to be obtained that can be used to create new classes of anti-SARS-CoV drugs and be potentially useful for screening drugs that inhibit the entry of other viruses into host cells.

In addition to inhibiting the membrane binding process, another way to prevent the virus from growing in host cells is to inhibit essential enzymes such as the SARS-CoV 3CL^pro^ and PL^pro^ viral cysteine proteases. Some natural compounds, including phenolics, indeed have this capacity, as will be discussed below.

In a study carried out by Lin et al. [60], the authors tested the capacity of some phenolic compounds to inhibit the 3C-like protease (3CL^pro^) of SARS-CoV. It is important to highlight that this protein mediates the proteolytic processing of replicase polypeptides 1a and 1ab into functional proteins and, therefore, it has become an important target for drug development. The authors identified aloe-emodin, an anthraquinone, and hesperetin, a flavanone, obtained from the roots of *Isatis indigotica* Fortune, as two compounds that inhibit the cleavage activity of the 3CL^pro^ in a dose-dependent way **(**Table 4). Ho et al. [61] also studied the anthraquinone emodin derived from *Rheum officinale* Baill. and *Polygonum multiflorum* Thunb., reporting that this significantly blocked the S protein and ACE2 interaction in a dose-dependent manner, suggesting that emodin may be considered a potential therapeutic agent in the treatment of SARS.

Park et al. [62] performed a biological evaluation on nine phlorotannins isolated from the edible brown algae *Ecklonia cava* Kjellman. The nine isolated phlorotannins, except phloroglucinol, possessed SARS-CoV 3CL^pro^ inhibitory activities in a dose-dependent and competitive manner. Among them, two eckol groups with a diphenyl ether linked, dieckol, showed the most potent SARS-CoV 3CL^pro^ trans/cis-cleavage inhibitory effects (IC_50_ = 2.7 and 68.1 μM, respectively). Moreover, dieckol exhibited a high association rate in the SPR (surface plasmon resonance) sensorgram and formed extremely strong hydrogen bonds to the catalytic dyad (Cys145 and His41) of the SARS-CoV 3CL^pro^. Park et al. [63] studied nine alkylated chalcones isolated from *Angelica keiskei* for their inhibitory activity against SARS-CoV proteases 3CL^pro^ and PL^pro^. Of the isolated alkylated chalcones, chalcone 6, containing the perhydroxyl group, exhibited the most potent 3CL^pro^ and PL^pro^ inhibitory activity with IC_50_ values of 11.4 and 1.2 µM. With this work, the authors proved that the chalcones exhibited competitive inhibition characteristics to the SARS-CoV 3CL^pro^, whereas noncompetitive inhibition was observed with the SARS-CoV PL^pro^. Park et al. [64] studied the inhibitory activity of *Broussonetia papyrifera*-derived polyphenols against 3CL^pro^ and PL^pro^ cysteine proteases. The authors obtained the isolated compounds broussochalcone B, broussochalcone A, 4-hydroxyisolonchocarpin, papyriflavonol A, 3′-(3-methylbut-2-enyl)-3′,4,7-trihydroxyflavane, kazinol A, kazinol B, broussoflavan A, kazinol F and kazinol J. All the studied polyphenols were more potent against PL^pro^ than against 3CL^pro^. Papyriflavonol A was the most potent inhibitor of PL^pro^ with an IC_50_ value of 3.7 μM (Table 4).

The lipophilic compounds tanshinones derived from *Salvia miltiorrhiza* Bunge were also found to be specific and selective inhibitors for the SARS-CoV 3CL^pro^ and PL^pro^ viral cysteine proteases (Park et al., 2012), the activity was significantly affected by subtle changes in structure. The IC_50_ values of these inhibitors, although higher than those of peptide-derived and small molecule viral cysteine protease inhibitors, were nonetheless in the low micromolar (from 0.8 to 30.0 μM).

Another class of compounds studied for anti-Sars-Cov-2 activity is the saikosaponins (triterpenoid glycosides). Cheng et al. [65] tested the anticoronaviral activity of saikosaponins A, B2, C and D and their mode of action. All these saikosaponins demonstrated antiviral activity in vitro, with the strongest activity being noted for saikosaponin B2. Furthermore, saikosaponin B2 also showed an inhibitory effect on viral attachment and penetration. In fact, Bahbah et al. [66] proposed saikosaponins A, B and D derived from *Bupleurum falcatum* L. (Umbelliferae) for the treatment of COVID-19. The authors stated in their published letter that a molecular docking study demonstrated that saikosaponin A has a high affinity to bind to a target receptor of the SARS-CoV-2, the ACE II receptor.

There are also studies testing the ability of natural extracts, instead of isolated compounds, to inhibit SARS-CoV-2 proteases. Luo et al. [67] tried to clarify the capacity of inhibition of the compounds from *Rheum palmatum* L. on the SARS-3CL protease. The authors verified that the hydroethanolic extracts from *R. palmatum* had a high level of anti-SARS-CoV 3CL protease activity. One of the extracts, RH121, obtained after extraction with ethyl acetate and separated by silica gel column chromatography with a gradient of chloroform/methanol (10:0-0:10, *v/v*), showed an IC_50_ value of 13.76 µg/mL and an inhibition rate up to 96%. Therefore, Luo et al. [67] concluded that the extracts from *R. palmatum* have a high level of inhibitory activity against 3CL protease, suggesting these may represent a potential therapeutic for SARS. Kim et al. [68] evaluated the effects of the ethanol extract of the seeds of *Psoralea corylifolia* L., which revealed high activity against the SARS-CoV PL^pro^ with an IC_50_ of 15 µg/mL. Given its potential, the authors fractionated the extract, obtaining six aromatic compounds, namely, bavachinin, neobavaisoflavone, isobavachalcone, 4′-*O*-methylbavachalcone, psoralidin and corylifol A. All the isolated flavonoids inhibited PL^pro^ in a dose-dependent manner with IC_50_ (concentration causing 50% of inhibition) ranging between 4.2 and 38.4 µM.

Finally, Schwarz et al. [69] based their research on the fact that the protein coded by the open-reading-frame 3a of SARS coronavirus forms a cation-selective channel that may become expressed in the infected cell, and the activity of the channel is involved in the mechanism of virus release. In this way, the authors tested the flavonols kaempferol, kaempferol glycosides and acylated kaempferol glucoside derivatives from the leaves of the plant *Quercus ilex* L. and kaempferol triglycoside from *Viola odorata* L. for their capacity to block the 3a channel and, therefore, inhibit virus release. The most effective one was the glycoside juglanin (carrying an arabinose residue) for inhibition of the 3a-mediated current, with an IC_50_ value of 2.3 µM. Kaempferol derivatives with rhamnose residue also seem to be quite effective (Table 4). 

**Table 4:** Anti-SARS coronavirus from phenolic and other compounds, type of extraction, extraction methodology and concentrations with activity.

Phenolic Compounds/Class	Type of Extract	Extraction Methods	Concentration	Reference
Tetra-*O*-galloyl-beta-*D*-glucose (TGG) Luteolin	Hydroethanolic extract	Herbs extracted by maceration with 85% ethanol at room temperature for 2 weeks	4.5 µM 83.4 µM	[59]
*Aloe emodin* Hesperetin	Aqueous extract from *Isatis indigotica* roots	Plant roots extracted twice with 10 volumes of distilled boiling water for 1 h	132 μM, 366 μM and 911.592 μM 60 μM, 8.3 μM and 2718 μM	[60]
Saikosaponins (A, B2, C and D)	Commercial standards	Saikosaponins dissolved in DMSO and further diluted with RPMI 1640 medium	25 µmmol	[70]
*A. emodin*	Aqueous extract of the root tuber from *Rheum officinale* Baill. and *Polygonum multiflorum* Thunb.	Deionized water	IC_50_ values ranged from 1 to 10 μg/mL.	[61]
Flavonoids from a Chinese multiherb remedy constituted by *Herba Houttuyniae, Flos Chrysanthemi* Indici, *Herba Artemisiae* Scopariae, *Herba Eupatorii* and *Fructus Tsaoko*	Hydroethanolic extract	Herb mixture extracted with 95% EtOH at room temperature	From the nine flavonoids with inhibitory effects, luteolin was the most potent with a CH_50_ (50% inhibitory concentration) of 0.19 mM	[71]
n.a.	Hydroethanolic extracts of *Rheum palmatum* L. (ethanol (75%))	Petroleum ether and chloroform, and ethyl acetate	Among the extracts, RH121 has the highest activity, with an IC_50_ of 13.76 μg/mL	[67]
Tanshinones	Hydroethanolic extract (95%) of the dried roots of *Salvia miltiorrhiza* Bunge	The crude extract was filtered and evaporated under reduced pressure. The obtained residue was suspended with distilled water	IC_50_ value of 0.7µM	[65]
Phlorotannins	Ethanol extract of brown alga *Ecklonia cava* Kjellman	Ethanol at room temperature	Of the nine phlorotannins tested, two eckol groups with a diphenyl ether linked dieckol showed the most potent SARS-CoV 3CL^pro^ trans/cis-cleavage inhibitory effects; IC_50_ = 2.7 and 68.1 μM, respectively)	[62]
Flavonoids (bavachinin, neobavaisoflavone, isobavachalcone, 4′-O-methylbavachalcone, psoralidin and corylifol A)	Fractionation of hydroethanolic extract from the seeds of *Psoralea corylifolia* L.	Ethanol, water and *n*-hexane	IC_50_ ranging between 4.2 and 38.4 µM	[68]
Juglanin Kaempferol-3-*O*-α-rhamnopyranosyl(1→2) [α-rhamnopyranosyl(1→6)]-β-glucopyranoside	Kaempferol acylated glucosides were previously isolated from polar extracts from the leaves of the plant *Quercus ilex* L.; the kaempferol triglycoside was an isolate from *Viola odorata* L (decoction and infusion)	Cyclohexane, Et_2_O, MeOH and MeOH–H_2_O	The most effective one was the glycoside juglanin with an IC_50_ of 2.3 µM	[69,72]
Alkylated chalcones	Hydroethanolic extract from the leaves of *Angelica keiskei* Ito	95% ethanol for a week at room temperature	IC_50_ values of 11.4 (3CL^pro^) and 1.2 µM (PL^pro^)	[63]
Broussochalcone B, Broussochalcone A, 4-hydroxyisolonchocarpin, Papyriflavonol A, 3′-(3-methylbut-2-enyl)-3′,4,7-trihydroxyflavane, Kazinol A, Kazinol B, Broussoflavan A, Kazinol F, Kazinol J	Ethanolic extracts from the roots of *Broussonetia papyrifera* (L.) Vent.	Ethanol at room temperature and evaporated using a rotary evaporator at temperatures below 45 °C to obtain the total extract.	Papyriflavonol A was the most potent inhibitor of PL^pro^ with an IC_50_ of 3.7 μM.	[64]
Epicatechin 5-*O*-beta-D-glucopyranoside-3-benzoate Neohesperidin Kaempferol-3,7-*O*-dirhamnoside (Kaempferitrin) Quercetin-3-*O*-neohesperidoside Oleic acid 3,4,5-Trimethoxyphenol Epicatechin Oxypeucedanin hydrate 3-Feruloylquinic acid Eriodictyol Apigenin Luteolin C-Methyl flavone β-amyrin Isovitexin-2″-*O*-rhamnoside	Methanolic extracts from *Fragaria ananassa* Duch.	80% methanol at room temperature.	Strawberry methanolic extract showed the highest antiviral activity against SARS-CoV-2 with an IC50 value to 0.0062 µg/mL	[2]
Sinapic acid	Ethanolic extract of broccoli (obtained from a local market)	95% ethanol	Potent SARS CoV-2 inhibition with a half-maximal inhibitory concentration (IC50) value of 2.69 µg/mL	[73]
Derivatives of luteolin, kaempferol, apigenin, isorhamnetin, myricetin, chrysoeriol, biochanin, isookanin and scutellarein	*V. vinifera* (var. Paulsen 1103) leaf extract	75% (*v*/*v*) methanol/0.05% (*v*/*v*) trifluoroacetic acid	Leaf extract was able to inhibit both HSV-1 and SARS-CoV-2 replication in the early stages of infection by directly blocking the proteins enriched on the viral surface, at a very low concentration of 10 µg/mL	[74]
TFC of sampled wild *S. nigra* was 9.57 ± 0.65 mg RE g^−1^ DW of plant material for berry extracts and 77.59 ± 10.23 mg RE g^−1^ DW of plant material for flower extracts. TPC of sampled wild *S. nigra* was 41.31 ± 9.44 mg gallic acid equivalent (GAE) g^−1^ DW for berry extracts and 451.72 ± 25.31 mg GAE g^−1^ DW for flower extracts	Ethanolic Extract of *Sambucus nigra* L. berry and flowers	80 % ethanol at 60 °C	Concentration-dependent inhibition of ACE2-SARS-CoV2 S-protein RBD binding was demonstrated in vitro for elderberry fruits and flowers extracts (IC50 of 1.66 mg DW ml^−1^ and 0.532 mg DW ml^−1^, respectively)	[75]
Caffeic acid, caftaric acid, chlorogenic acid, cichoric acid, cynarin, echinacoside	*Echinacea purpurea* (echinaforce)	65% ethanol	50 µg/mL inactivation of SARS-CoV1 and 2	[76]
Glyzyrrhizin	*Glycyrrhiza glabra*	Acquired compound	300 mg/L	[77]
Baicalein	*Scutellaria baicalensis*	70% ethanol (crude extract of plant) and commercial baicalein	IC50 8.52 µg/mL (crude extract) and 0.39 µg/mL (baicalein)	[78]
Epigallocatechin, genistein, sulforaphane, chlorogenic acid, resveratrol and quercetin	-	Acquired compounds	IC_50_—33.9 µg/mL (epigallocatechin)	[79]
Whole plant extract	*Taraxacum officinale*	Aqueous extractions for 1 h	EC_50_—14.9 mg/mL	[80]

The latest reviews demonstrate that extracts or preparations with inhibitory activity against coronaviruses include *Lycoris radiata* (L’Hér.) Herb., *Artemisia annua* L., *Pyrrosia lingua* (Thunb.) Farw., *Lindera aggregata* (Sims) Kosterm. and *Isatis indigotica* Fortune, and the extracts of *Rheum officinale* Baill., *Polygonum multiflorum* Thunb., *Houttuynia cordata* Thunb., *Gentiana scabra* Bunge, *Dioscorea batatas* L., *Cassia tora* L., *Taxillus chinensis* (DC.) Danser, *Cibotium barometz* (L.) J.Sm., *Anthemis hyaline* DC., *Nigella sativa* L. and *Citrus sinensis* (L.) Osbeck [45]. Furthermore, regarding the class of bioactive compounds that act against coronaviruses, alkaloids, phenolic compounds including flavonoids, chalcones, lignins and tannins, glycosides, stand out as the most promising agents [45].

## 6. Coronavirus Inhibition Assays

Among the most used methods to find coronavirus inhibitors, high-throughput screening stands out. This research technique, mainly used in drug discovery as a practical and rapid approach, allowed the finding of potential inhibitors of virus replication. In a study carried out by Touret et al. [81], the authors proceeded with the reuse of approved drugs, a strategy that can bypass the lengthy stages of drug development. In this study, the authors screened an FDA-authorized chemical library composed of 1520 approved drugs, identifying those that had already demonstrated an antiviral effect in vitro against SARS-CoV-2, inhibiting its replication. Thus, 90 compounds were identified as positive screening results and were grouped according to their chemical composition and known therapeutic effect.

Afterwards, EC_50_ and CC_50_ (the concentration that reduces 50% of the total cell number) values were determined for a subset of 15 compounds. From the antiviral agents that showed better results, it was possible to highlight macrolides antibiotics, proton pump inhibitors, antiarrhythmic agents, or central nervous system drugs, with EC_50_ values between 2 and 20 µM. Brown et al. [82] performed a high-throughput screening for inhibitors of the SARS-CoV-2 protease using a FRET (fluorescence resonance energy transfer)-biosensor. Protein-based biosensors allow screening to be performed using routine molecular laboratory equipment, not requiring expensive reagents. In this work, the authors presented a biosensor for the 3CL^pro^ cysteine protease of SARS-CoV-2, comprising a FRET-capable pair of fluorescent proteins held in proximity by a protease-cleavable ligand. The usefulness of this biosensor was demonstrated for inhibitor discovery by screening 1280 compounds from the “Library of Pharmaceutically Active Compounds” collection. The screening identified 65 inhibitors, with the 20 most active exhibiting sub-micromolar inhibition of 3CL^pro^ in follow-up EC_50_ assays.

A study performed by Jin et al. [83] showed the results of a program that aimed to rapidly discover lead compounds for clinical use by combining structure-assisted drug design, virtual drug screening and high-throughput screening. This program was focused on identifying drugs that target the M^pro^ protease of SARS-CoV-2, a coronavirus enzyme with a critical role in mediating viral replication and transcription. The authors identified a mechanism-based inhibitor (N3) through computer-aided drug design and then determined the crystal structure of SARS-CoV-2 M^pro^ in complex with this compound. Through a combination of structure-based and high-throughput virtual screening, they screened more than 10,000 compounds, including approved drugs, clinical trial drug candidates and other pharmacologically active compounds, as M^pro^ inhibitors. Among six compounds with positive results, “ebselen” showed promising antiviral activity in cell-based assays. This work demonstrated the effectiveness of the screening strategy used, which can lead to the rapid discovery of drugs with clinical potential in response to new infectious diseases. In addition to these high-throughput screening inhibition assays, other assays have also emerged in the constant search for SARS-CoV-2 inhibitors.

Effectively, when searching for SARS-CoV-2 inhibition assays, several high-throughput screening studies emerge, most of which involve cell-based assays [34,84,85,86,87]. However, it is also possible to find studies that use, for example, virological tools. Nie et al. [88] developed a pseudovirus neutralization assay for SARS-CoV-2. This methodology was based on a VSV pseudovirus production system. *Vesicular stomatitis Indiana* virus (VSIV or VSV), commonly known as *Indiana vesiculovirus*, is widely used at the laboratory level to study viral evolution. Therefore, based on a VSV pseudovirus production system, the authors developed a pseudovirus-based neutralization assay to evaluate neutralizing antibodies against SARS-CoV-2. The main parameters for this assay were optimized, including cell types, cell number and virus inoculum. When tested against the SARS-CoV-2 pseudovirus, sera from convalescent SARS-CoV-2 patients showed high neutralizing potency, reinforcing its potential as a therapeutic.

Govinda et al. [89] developed a suite of computational models, designed as REDIAL-2020, for estimating small molecule activities in a range of SARS-CoV-2-related assays. Models were trained using publicly available high-throughput screening data and by employing different descriptor types and various machine-learning strategies. The authors described the development and use of 11 models covering viral entry, viral replication, live virus infectivity, in vitro infectivity, and human cell toxicity. REDIAL-2020 is available as a web application through the DrugCentral web portal (http://drugcentral.org/Redial, accessed on 15 December 2022) and can serve as a quick online tool to identify active molecules for COVID-19 treatment.

The above-mentioned assays are the primarily available ones found in the literature and have as principal targets the central proteases of SARS-CoV-2 involved in the virus replication (3CL^pro^ and M^pro^). However, it should be considered that strategies other than virus replication are in place to find SARS-CoV-2 inhibitors based on different parameters. As the entry of pathogenic viruses into susceptible cells is the first step in the infection process, blocking this initial stage has been chosen as one of the main strategies of the therapeutics under development.

Like the other coronaviruses, SARS-CoV-2 uses the homotrimeric spike glycoprotein on the envelope to bind to its cellular receptors and starts the first stage of its lifecycle. Such binding triggers a cascade of events, leading to the fusion between cell and viral membranes and allowing cell entry [90].

The spike protein has two domains; S1 is responsible for ACE2 recognition, the recently identified host cell receptor, and S2 mediates membrane fusion [91]. Although a complex process, as it encompasses multiple steps with numerous interactions, several studies have emerged trying to design entry inhibitors against coronaviruses.

Therefore, and based on SARS-CoV-2 spike protein recognition of the ACE2 host cell receptor with its Receptor Binding Domain (RBD), some studies propose an intervention at the RBD-ACE2 interface to potentially stop the infection process [92].

Although many of the studies performed with coronaviruses essentially use designed peptides and proteins or antibodies, some work is carried out using small molecules [1,93].

These are still preferred for drug development given their improved pharmacokinetics, stability and dosing logistics compared to proteins or peptides [94,95]. Although some natural compounds have already been tested against SARS-CoV-2, their mechanism of action remains mostly unknown. However, some studies suggest that they can act on preventing virus entry into the host cell. Milewska et al. [96] synthesized several polymer-based compounds showing prominent anticoronaviral activity. Two of the tested compounds, designed as HTCC (compound 10) and HM-HTCC (compound 11), inhibited HCoV-NL63 (Human coronavirus NL63), blocking the interaction of the virus with its ACE2 receptor and, therefore, avoiding viral entry. Some phenolic compounds, particularly flavonoids, have also shown inhibitory activity against coronavirus, namely, luteolin and quercetin. The authors verified that luteolin could bind avidly to the SARS-CoV S2 protein and inhibit viral entry of SARS-CoV into Vero E6 cells. Both luteolin and quercetin exhibited antiviral effects against SARS-CoV, with IC_50_ values of 10.6 μM and 83.4 μM, respectively [59,97]. Other recent studies demonstrated by in silico assays that stilbene-based compounds in general and resveratrol, in particular, can be promising anti-COVID-19 drug candidates acting through disruption of the spike protein, i.e., they have inhibitory activity against ACE2 receptor [98].

In 2021, researchers developed a multivalent coronavirus vaccine by using epitopes of the spike protein connected to a cowpea mosaic virus as a vector. When tested in vitro, the soluble solutions of the vaccine showed high neutralizing power, although when administered to mice they did not neutralize the virus. Overall, this strategy, when further improved may help fight the pandemic due to a higher temperature resistance of plant viruses [99].

## 7. Future and Trends

The relationship between viruses and humans has always been about balancing infectiousness and lethality, and while outbreaks of highly contagious and lethal viruses have been few, recent studies have shown that they are expected to increase in coming years. Improvement of hygiene and knowledge of viruses has helped mankind to protect itself from viruses, but some pockets of densely packed population and wild animals has created the perfect concoction for spillover of viruses and other pathogens from animals to humans, and while this could be relatively easy to overcome, other changes are more cumbersome.

Climate change, which represents a huge issue on its own [100], is probably the most important factor that will increase the pressure for viruses such as the Coronaviridae to jump from animals to humans [101]. Deforestation increases pressure on wild ecosystems, making wild animals share habitats with humans. Beyond this, floods increase the prevalence of viral vectors, and droughts change ecosystems, making them abruptly shift, allowing for new connections between animals, resulting in a mixture of viruses that can easily infect humans.

Beyond the grim scenario of climate change helping to increase the prevalence of viruses, the unknown long-standing symptoms, and opportunistic diseases in patients previously infected with COVID-19 is another matter of concern. Autoimmune diseases are manifesting themselves in some patients after recovering from mild to severe COVID-19, which shows the long road ahead for satisfactory knowledge of this illness and its sequelae over time [102,103,104].

While mitigating climate change may help reduce the probability of higher occurrences of a global pandemic, it will not avoid them. In fact, it is hardly possible to completely avoid contained outbreaks of viral pandemics, and thus, mankind can only work to reduce their widespread and be prepared to quickly implement vaccines, treatments and measures to mitigate their effects, which could control or even preview further outbreaks. These efforts must be carried out by all branches of society, and all possibilities should be pondered.

Plants, being a widely disseminated and endless resource, and are in some cases the cure or treatment for many previous diseases and pathogenic vectors, are once again paramount to fend of this coronavirus, and will be effective in the coming pandemics.

The use of plant extracts as adjuvants in antiviral treatments, as accessory treatment for symptomatic relief, as application in protective equipment for health professionals, and being mixed into disinfectant solutions and other applications is still being studied, and results of publications and intellectual property are expected to be rolled out in the coming months and years.

## Figures and Tables

**Table 2:** Approved and under rolling review of vaccines in the EU and the US.

Vaccine	Type	Manufacturer	Approval
Approved			
Comirnaty	mRNA	Pfizer-BioNTech	EU and US
Spikevax (former Moderna)	mRNA	Moderna	EU and US
Vaxzevria (former AstraZeneca)	Modified adenovirus	AstraZeneca	EU
Janssen	Modified adenovirus	Janssen	EU and US
Novavax	Spike glycoprotein subunit	Novavax	EU and US
Under Rolling Review			
Sanofi (Vidprevtyn)	Spike glycoprotein subunit	Sanofi-GSK	Sought in EU
HIPRA Human Health	Spike glycoprotein subunit	HIPRA	Sought in the EU
Valneva	Inactivated coronavirus	Valneva	Sought in the EU

Sources: https://commission.europa.eu/strategy-and-policy/coronavirus-response/safe-covid-19-vaccines-europeans_en (accessed on 15 December 2022) https://www.fda.gov/emergency-preparedness-and-response/coronavirus-disease-2019-covid-19/covid-19-vaccines (accessed on 15 December 2022).

## Data Availability

No new data was produced.
